# 5-α reductase inhibitors, benign prostatic hyperplasia, and risk of male breast cancer

**DOI:** 10.1007/s10552-015-0622-4

**Published:** 2015-06-25

**Authors:** David Robinson, Hans Garmo, Lars Holmberg, Pär Stattin

**Affiliations:** Department of Surgery and Perioperative Sciences, Urology and Andrology, Umeå University, 901 85 Umeå, Sweden; Department of Urology, Ryhov County Hospital, 551 85 Jönköping, Sweden; Regional Cancer Centre, Uppsala University Hospital, 751 83 Uppsala, Sweden; King’s College London, Medical School, Division of Cancer Studies, Cancer Epidemiology Group, London, WC2R 2LS UK

**Keywords:** 5-α reductase inhibitors, Male breast cancer, Benign prostatic hyperplasia

## Abstract

**Purpose:**

5-α reductase inhibitors (5-ARI) have been suggested to increase the risk of male breast cancer. The aim of this study was to study the risk of breast cancer in men on 5-ARI, in men with benign prostatic hyperplasia (BPH) not on 5-ARI, and in men without BPH.

**Methods:**

We performed a population-based cohort study in Sweden with data from The Prescribed Drug Register, The Patient Register, and The Cancer Register. Men on 5-ARI, men on α-blockers, or men who had undergone a transurethral resection of the prostate (TUR-P) prior to or during 2006–2008 were included as exposed to BPH and a specific treatment thereof. For each exposed man, five unexposed men were selected. Risk of breast cancer was calculated in Cox proportional hazard models.

**Results:**

There were 124,183 exposed men and 545,293 unexposed men, and during follow-up (median 6 years), 99 men with breast cancer were diagnosed. Compared to unexposed men, men on 5-ARI had a hazard ratio (HR) of breast cancer of 0.74 (95 % confidence interval (CI) 0.27–2.03), men on α-blockers had HR 1.47 (95 % CI 0.73–2.95), and men with a TUR-P had HR 1.99 (95 % CI 1.05–3.75).

**Conclusion:**

No increased risk of breast cancer was observed for men on 5-ARI. However, the increased risk of breast cancer among men who had undergone a TUR-P, a strong indicator of BPH, suggests that the endocrine milieu conducive to BPH is associated with male breast cancer.

## Introduction

5-α reductase inhibitors (5-ARI) are commonly used for the treatment of lower urinary tract symptoms (LUTS) often caused by benign prostatic hyperplasia (BPH). The mode of action of 5-ARI is to inhibit the conversion of testosterone to dihydrotestosterone (DHT), the most potent androgen in the prostate [1]. This inhibition leads to a decreased prostate volume, and 5-ARI thereby reduces LUTS, the risk of acute urinary retention, and the risk of prostate surgery [2, 3]. Side-effects of 5-ARI include decreased libido, erectile dysfunction, and reduced ejaculatory volume [2, 4]. In addition, men on 5-ARI may have an increased risk of breast cancer. Patient information issued by the US Food and Drug Administration (FDA) for finasteride includes the following statements: “In rare cases male breast cancer has been reported” and “The relationship between long-term use of finasteride and male breast neoplasia is currently unknown” [5]. These statements were based on a few sporadic cases of breast cancer in men on 5-ARI in two clinical trials and on spontaneous reports to the FDA [3, 6, 7]. In two recent case–control studies, no association between 5-ARI and breast cancer was found [8, 9].

The aim of this study was to assess the risk of breast cancer in men on 5-ARI and in men with BPH not treated with 5-ARI using data from nationwide, population-based healthcare registers and demographic databases in Sweden.

## Materials and methods

This investigation was conducted as a cohort study in the male population in Sweden with study men identified from 1 January 2006 to 31 December 2008. All men with a filled prescription for a 5-ARI or an α_1_-adrenergic receptor blocker (α-blocker), or who had undergone a transurethral resection of the prostate (TUR-P) prior to or during the study period, were included. The use of α-blockers, another class of drugs for the treatment of LUTS that block receptors in the smooth muscle in the prostate, and the receipt of a TUR-P were used as indicators of BPH. For each man with an exposure to 5-ARI, α-blocker, or a TUR-P, five unexposed men of same age (±1 year) as the exposed man in each subgroup were randomly selected from the same county of residence. This was done by identifying men without these exposures in the Register of the Total Population and Population Changes held at Statistics Sweden. Unexposed men were required to be alive at the index date of the exposed.

We were not able to find five unexposed men from the same county and in the same age for all men, so some men have less than five controls. Follow-up started at 1 January 2006 for men exposed prior to that date, and for men who started exposure later, follow-up started at the date of surgery or first filled prescription. For unexposed men, follow-up started at the same date as their index man. No men with prevalent prostate cancer were included, and if a prostate cancer was detected during follow-up, this was ignored. Follow-up ended at the date of diagnosis of breast cancer, date of emigration, date of death, date of androgen deprivation therapy for prostate cancer, or 31 December 2011, whichever date came first.

### Data sources for exposure, end-point, and covariates

Data on filled prescriptions for the 5-ARI finasteride and dutasteride [Anatomic Therapeutic Chemical classification system (ATC) code G04CB] and all types of α-blockers (G04CA01 to 4) used for the treatment of LUTS were extracted from The Prescribed Drug Register, which includes all prescriptions dispensed since July 2005 in Sweden [10]. We also retrieved information on antidiabetic drugs (insulin and peroral drugs) as an indicative for a diagnosis of diabetes mellitus and cholesterol-lowering agents (statins) indicative of hypercholesterolemia, since these are associated with male breast cancer [11, 12]. The Prescribed Drug Register contains data on the amount and dose of each drug as well as the date of prescription and dispensing.

As of 1987, the National Patient Register collects information on inpatient care including surgical procedures, and discharge diagnoses coded according to International Classification of Diseases (ICD-9 and subsequently ICD-10) [13]. From this register, we obtained information on the use of TUR-P in the study population and conditions known or assumed to be associated with male breast cancer including Klinefelter’s syndrome [14], gynecomastia [14], and liver disease with the definitions used by Charlson [15, 16]. Men with breast cancer (ICD C50) were identified from the Swedish Cancer Register. We assessed comorbidity for all study participants. The Longitudinal Integration Database for Health Insurance and Labour Market Studies (LISA) is a nationwide demographic database with data on socioeconomic factors. From this database, we obtained information on educational level, which was categorized as low ≤9 years of school, middle 10–12 years, and high ≥13 years, which in Sweden corresponds to mandatory school, high school, and college or university, and we used education level as an adjustment in the analyses [17].

### Statistical analysis

We performed two Cox proportional hazard models [18] with the results presented as hazard ratios (HR) with 95 % confidence intervals (CI), and in both analyses, we used age as a time-dependent covariate.

First we estimated the risk of breast cancer in relation to exposures determined at study entry, α-blockers, TUR-P, or 5-ARI compared to non-exposed men. No adjustments for potential confounders were made in this analysis, and subsequent changes in exposures were ignored.

In the second analysis, subsequent exposures to α-blockers, TUR-P, or 5-ARI were taken into account using time-dependent covariates. This analysis was performed as both a univariable and a multivariable analysis, including potential confounders such as the use of antidiabetic drugs and statins. This second analysis was first performed for (A) the full study group, (B) men unexposed at entry to the study, (C) men exposed to α-blockers/TUR-P at entry to the study, and (D) men exposed to 5-ARI at entry to the study. Stratification was done in order to assess all potential associations between exposure to BPH and male breast cancer. We performed a sensitivity analysis where all men with a prostate cancer diagnosis during follow-up were censored. Third, we studied the time between the start of 5-ARI exposure and the date of breast cancer diagnosis. Finally, we performed a meta-analysis of the current study and the two previous studies on 5-ARI and breast cancer.

## Results

In total, 669,476 men were included in the study, with a mean age of 70 years at the study start for non-exposed men, 66 years for men on α-blockers, 74 years for men who had undergone TUR-P, and 72 years for men on 5-ARI (Table 1). A majority of men (73 %) were included in the study on 1 January 2006 with an ongoing exposure to α-blockers or 5-ARI, or a history of TUR-P. The median follow-up was 6 years. Prevalence of diabetes and hypercholesterolemia was quite high: 10 % of the men were on antidiabetic drugs and almost 20 % on statins. Gynecomastia, liver disease, and Klinefelter’s syndrome were rare in the study population, and there were no men with breast cancer after 5-ARI exposure, so these variables could be not included in the multivariable model.

**Table 1:** Baseline characteristics and follow-up according to exposure at the start of the study period

	Unexposed (*n* = 545,293)	α-Blocker (*n* = 53,267)	TUR-P (*n* = 34,296)	5-ARI (*n* = 36,620)	All (*n* = 669,476)
Age at study start, Mean (SD)	69.6 (11.7)	65.7 (12.0)	73.8 (9.2)	72.0 (11.4)	69.6 (11.7)
Median follow-up (Q1–Q3, years)	6.0 (4.1–6.0)	5.8 (4.0–6.0)	6.0 (4.5–6.0)	6.0 (3.9–6.0)	6.0 (4.1–6.0)
Start of follow-up, *n* (%)					
31 December 2005	395,614 (72.6)	32,881 (61.7)	33,570 (97.9)	29,265 (79.9)	491,330 (73.4)
2006	75,774 (13.9)	7,066 (13.3)	303 (0.9)	2,685 (7.3)	85,828 (12.8)
2007	36,903 (6.8)	6,794 (12.8)	233 (0.7)	2,263 (6.2)	46,193 (6.9)
2008	37,002 (6.8)	6,526 (12.3)	190 (0.6)	2,407 (6.6)	46,125 (6.9)
Gynecomastia, *n* (%)					
No	545,219 (100.0)	53,253 (100.0)	34,290 (100.0)	36,613 (100.0)	669,375 (100.0)
Yes	74 (0.0)	14 (0.0)	6 (0.0)	7 (0.0)	101 (0.0)
Liver disease, *n* (%)					
No	541,686 (99.3)	52,828 (99.2)	34,088 (99.4)	36,412 (99.4)	665,014 (99.3)
Mild	2,894 (0.5)	372 (0.7)	169 (0.5)	173 (0.5)	3,608 (0.5)
Moderate or severe	713 (0.1)	67 (0.1)	39 (0.1)	35 (0.1)	854 (0.1)
Klinefelter’s syndrome, *n* (%)					
No	545,258 (100.0)	53,259 (100.0)	34,293 (100.0)	36,619 (100.0)	669,429 (100.0)
Yes	35 (0.0)	8 (0.0)	3 (0.0)	1 (0.0)	47 (0.0)
Statins, *n* (%)					
No	441,431 (81.0)	41,279 (77.5)	27,176 (79.2)	27,252 (74.4)	537,138 (80.2)
Yes	103,862 (19.0)	11,988 (22.5)	7,120 (20.8)	9,368 (25.6)	132,338 (19.8)
Diabetes mellitus, *n* (%)					
No DM	493,189 (90.4)	48,002 (90.1)	30,504 (88.9)	32,527 (88.8)	604,222 (90.3)
Peroral drugs	34,558 (6.3)	3,690 (6.9)	2,381 (6.9)	2,852 (7.8)	43,481 (6.5)
Insulin	17,546 (3.2)	1,575 (3.0)	1,411 (4.1)	1,241 (3.4)	21773 (3.3)
Education level, *n* (%)					
High	102,220 (18.7)	12,774 (24.0)	5,529 (16.1)	7,886 (21.5)	128,409 (19.2)
Middle	195,070 (35.8)	20,742 (38.9)	11,619 (33.9)	13,036 (35.6)	240,467 (35.9)
Low/missing	248,003 (45.5)	19,751 (37.1)	17,148 (50.0)	15,698 (42.9)	300,600 (44.9)

α-Blocker ATC code G04CA01 to 4

*5-ARI* 5-α reductase inhibitors ATC code G04C, *TUR-P* transurethral resection of the prostate

Q1–Q3 = Lower quartile and upper quartile

**Fig. 1:**
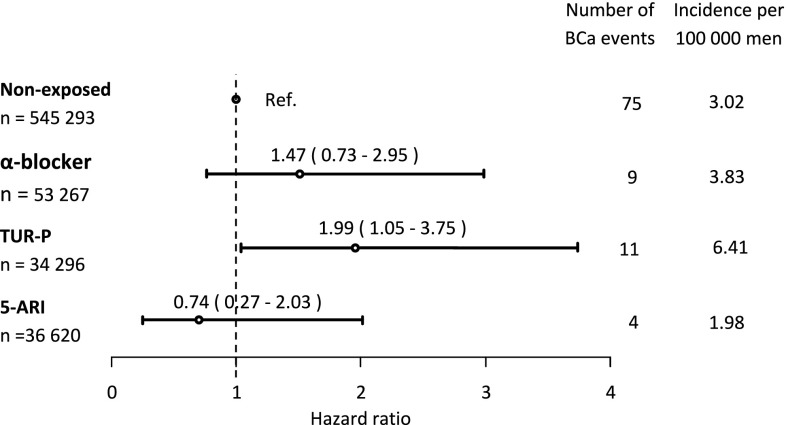
Hazard ratio of breast cancer according to exposure at the study start. Hazard ratio and 95 % confidence intervals of breast cancer according to exposure to α-blocker, TUR-P, or 5-ARI at the start of the study period with unexposed as reference. *TUR-P* transurethral resection of the prostate, *5-ARI* 5-α reductase inhibitor

During the study period, 99 men were diagnosed with breast cancer, with a mean age of 78 years (standard deviation ± 9.0 years). Compared to the unexposed, men on 5-ARI had a non-significant decrease in the risk of breast cancer, HR 0.74 (95 % CI 0.27–2.03); men on α-blockers had a non-significant increase, HR 1.47 (95 % CI 0.73–2.95); and men who had undergone a TUR-P had a significantly increased risk, HR 1.99 (95 % CI 1.05–3.75) (Fig. 1).

In the Cox multivariable analysis, the risk of breast cancer was not increased for men who started 5-ARI during the study period compared to men who started α-blockers or received a TUR-P during the study period in the full study group and in all subgroups according to exposure at the study start (Table 2). For example, in the full study group, men exposed to 5-ARI had a non-significantly lower risk of breast cancer, HR 0.65 (95 % CI 0.32–1.31). In men who were non-exposed at the study start, those who started 5-ARI during the study period had the same risk of breast cancer as men who did not start 5-ARI, HR 0.96 (95 % CI 0.32–2.85). In contrast, among initially unexposed men, those who subsequently started to use α-blockers or underwent TUR-P had an insignificantly increased risk, HR 1.86 (95 % CI 0.84–4.13). The risk estimates were essentially unaltered after adjustment for known risk factors for male breast cancer, and the increase in the risk of breast cancer for men on oral antidiabetic drugs was not significant, HR 1.40 (95 % CI 0.68–2.87). When men with a prostate cancer detected during follow-up were censored, the results from the time-updated analysis were unchanged (data not shown).

**Table 2:** Hazard ratio of breast cancer in Cox regression analysis in relation to time-updated covariates according to exposure to α-blocker, TUR-P, or 5-ARI and unexposed at the start of the study period and subsequent additional exposure

Treatment initiated during study period	Number of cases of breast cancer	Incidence per 100,000 person-years	Univariable analysis		Multivariable analysis	
			HR	95 % CI	HR	95 % CI
A. Full study group						
α-Blocker/TUR-P						
No α-blocker or TUR-P	69	2.59	1.00	Ref.	1.00	Ref.
α-Blocker or TUR-P	30	4.77	1.76	(1.15–2.71)	1.85	(1.19–2.87)
5-ARI						
No 5-ARI	90	3.02	1.00	Ref.	1.00	Ref.
5-ARI	9	2.88	0.81	(0.41–1.61)	0.65	(0.32–1.31)
DM						
No DM	85	2.93	1.00	Ref.	1.00	Ref.
DM—peroral	11	3.90	1.23	(0.65–2.30)	1.13	(0.59–2.14)
DM—insulin	3	2.84	0.89	(0.28–2.81)	0.81	(0.26–2.60)
Statin						
No Statin	62	2.76	1.00	Ref.	1.00	Ref.
Statin	37	3.84	1.35	(0.89–2.04)	1.32	(0.86–2.02)
Education level^a^						
High	16	2.45	1.00	Ref.	1.00	Ref.
Middle	41	3.41	1.28	(0.71–2.27)	1.27	(0.71–2.27)
Low/missing	42	2.93	0.86	(0.48–1.55)	0.87	(0.49–1.56)
B. Non-exposed at study start						
α-Blockers/TUR-P						
No α-blocker or TUR-P	67	2.63	1.00	1.00 (Ref.)	1.00	Ref.
α-Blocker or TUR-P	8	5.88	1.84	(0.88–3.85)	1.86	(0.84–4.13)
5-ARI						
No 5-ARI	71	2.74	1.00	(Ref.)	1.00	Ref.
5-ARI	4	4.48	1.28	(0.46–3.51)	0.96	(0.32–2.85)
DM						
No DM	63	2.66	1.00	Ref.	1.00	Ref.
DM—peroral	9	3.99	1.39	(0.69–2.80)	1.40	(0.68–2.87)
DM—insulin	3	3.52	1.22	(0.38–3.89)	1.24	(0.38–4.00)
Statin						
No Statin	52	2.71	1.00	Ref.	1.00	Ref.
Statin	23	3.01	1.02	(0.62–1.69)	0.95	(0.57–1.59)
Education level^a^						
High	9	1.73	1.00	Ref.	1.00	Ref.
Middle	32	3.28	1.77	(0.84–3.70)	1.76	(0.84–3.69)
Low/missing	34	2.87	1.27	(0.60–2.67)	1.27	(0.60–2.66)
C. α-Blocker/TUR-P at study start						
5-ARI						
No 5-ARI	19	4.93	1.00	Ref.	1.00	Ref.
5-ARI	1	2.14	0.42	(0.06–3.11)	0.37	(0.05–2.79)
DM						
No DM	18	4.75	1.00	Ref.	1.00	Ref.
DM—peroral/insulin	2	3.74	0.71	(0.17–3.09)	0.53	(0.12–2.33)
Statin						
No Statin	8	2.71	1.00	Ref.	1.00	Ref.
Statin	12	8.76	3.51	(1.39–8.82)	3.76	(1.48–9.53)
Education level^a^						
High	6	6.53	1.00	Ref.	1.00	Ref.
Middle	7	4.33	0.57	(0.19–1.71)	0.57	(0.19–1.69)
Low/missing	7	3.95	0.35	(0.12–1.06)	0.36	(0.12–1.09)
D. 5-ARI at study start^b^						
α-Blocker/TUR-P						
No α-blocker/TUR-P	2	1.72	1.00	Ref.	1.00	Ref.
α-Blocker/TUR-P	2	3.32	2.00	(0.28–14.3)	1.99	(0.28–14.2)
Statin						
No Statin	2	1.76	1.00	Ref.	1.00	Ref.
Statin	2	3.19	1.50	(0.21–10.8)	1.53	(0.21–10.9)
Education level^a^						
High	1	2.52	1.00	Ref.	1.00	Ref.
Middle	2	3.13	1.10	(0.10–12.1)	1.09	(0.10–12.1)
Low/missing	1	1.37	0.40	(0.02–6.44)	0.40	(0.03–6.48)

*5-ARI* 5-α reductase inhibitor, *TUR-P* transurethral resection of the prostate, *HR* hazard ratio, *95* *% CI* confidence interval

^a^Not time-updated

^b^No breast cancers among men with DM

A) All 99 men in the full study group who were diagnosed with breast cancer during follow-up

B) 75 men with no exposure indicative of BPH at the start of the study period who were diagnosed with breast cancer during follow-up

C) 20 men exposed to α-blockers/TUR-P at the start of the study period who were diagnosed with breast cancer during follow-up

D) 4 men exposed to 5-ARI at the start of the study period who were diagnosed with breast cancer during follow-up

**Fig. 2:**
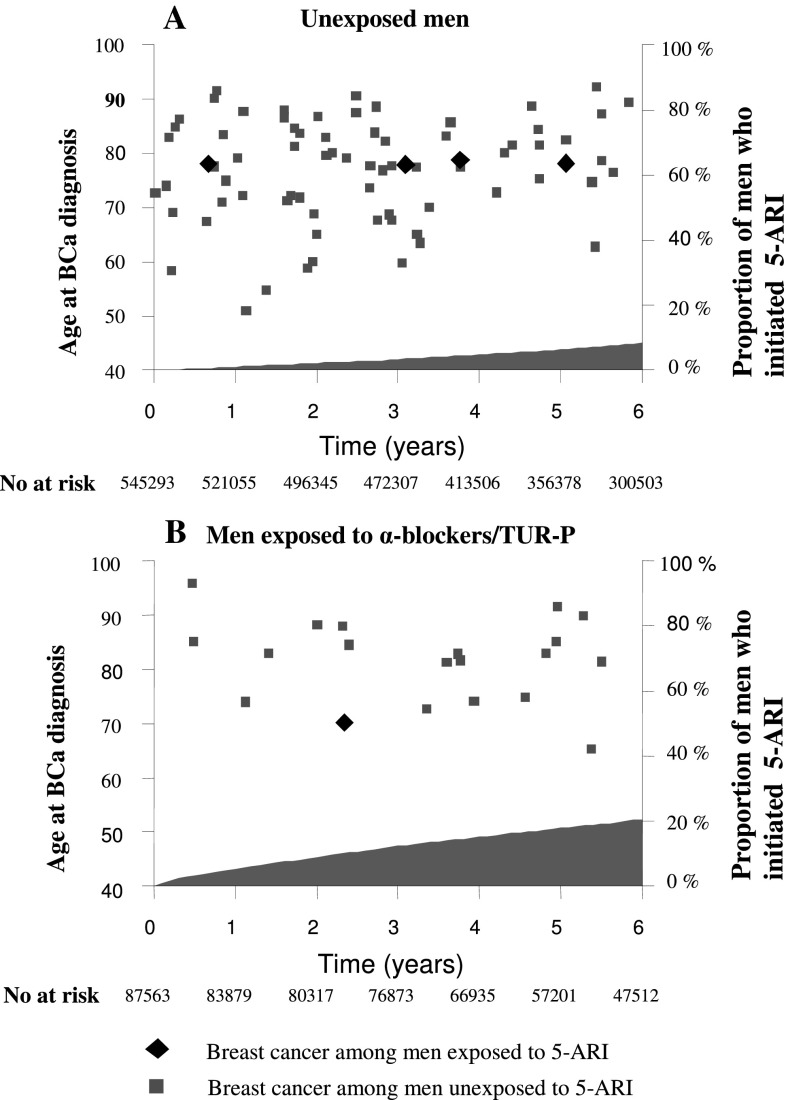
Time between the study start and the date of diagnosis of breast cancer and proportion of men who initiated 5-ARI treatment during the study period among (**a**) men unexposed to α-blockers or TUR-P as indicator of benign prostatic hyperplasia (BPH) before the start of the study period and number of men with a subsequent exposure to 5-ARI and (**b**) men exposed to α-blockers or TUR-P as indicator of BPH before study start and number of men with a subsequent exposure to 5-ARI. *BCa* breast cancer, *5-ARI* 5-α reductase inhibitor, *TUR-P* transurethral resection of the prostate

Figure 2 depicts the time between the study start and the date of breast cancer diagnosis, age at diagnosis, and the proportion of men who started 5-ARI during the study period. There was no association between the duration of 5-ARI exposure and occurrence of breast cancer, or any material difference in age between men with a subsequent exposure to 5-ARI and unexposed men.

Out of the 9 cases of breast cancer, 6 cases were diagnosed in men who had been exposed less than 3 years and 3 cases were diagnosed in men who had been exposed more than 3 years (data not shown).

**Fig. 3:**
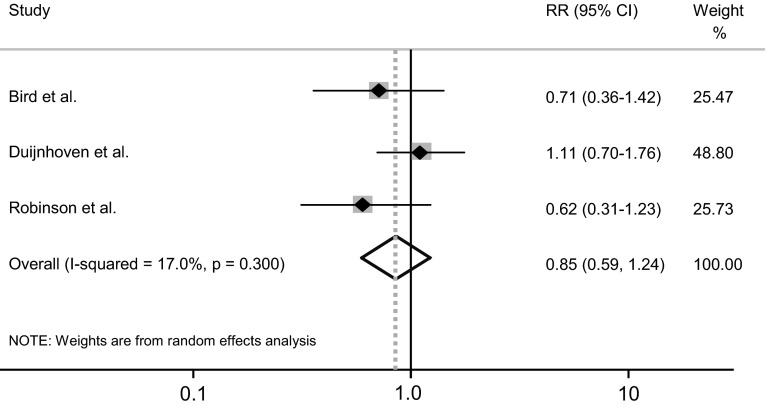
Meta-analysis of studies on 5-ARI and male breast cancer including the studies by Bird et al. [8], Duijnhoven et al. [9], and the current study

In the meta-analysis, we found an unaltered risk of male breast cancer, RR 0.85 (95 % CI 0.59–1.24) (Fig. 3).

## Discussion

In this nationwide, register-based, and population-based study, men on 5-ARI had a non-significant decrease in risk of breast cancer, whereas men on α-blockers had a non-significant increase and men who had received a TUR-P had a significantly increased risk of breast cancer.

Strengths of our study include the very high capture rate of the entire male population of Sweden in all registers that were used. The Prescribed Drug Register has an extremely high capture rate with less than 0.3 % incomplete records, and the Patient Register has a high capture rate for inpatient surgery [10, 13]. Limitations of our study include the unknown duration of exposure to 5-ARI and α-blockers before the study period for a majority of men due to the fact that The Prescribed Drug Register started in July 2005. The maximum follow-up was 6 years, which is in the range of those in previous studies [2, 3, 7]. Furthermore, we had no information on some known risk factors for male breast cancer such as family history, obesity, and exposure to radiation [19]. Even if we studied all men in Sweden exposed to 5-ARI during 3 years, a limitation to this study is the small number of cases with male breast cancer.

Male breast cancer is a rare disease with an annual incidence rate of 1 per 100,000 men [20], and previous clinical trials have reported a maximum of four men with breast cancer. For example, the “Medical therapy of prostatic symptoms” (MTOPS) study reported that four men on 5-ARI and no man in the placebo group were diagnosed with breast cancer [3], in “Proscar long term efficacy and safety study” (PLESS) none in the 5-ARI arm and two men on placebo were diagnosed with breast cancer [2], and in the Prostate cancer prevention trial (PCPT) one man with breast cancer was found in each of the 5-ARI and placebo arms [7]. Out of all men with breast cancer in the studies to date, 99 cases in the current study and 339 and 398 cases in the recent case–control studies by Bird et al. and Duijnhoven et al. [8, 9], only a small fraction of cases (34/836, 4 %) had been exposed to 5-ARI before the date of breast cancer diagnosis. Thus, very large pooled analyses will be needed to clarify whether there is any association between exposure to 5-ARI and male breast cancer at all.

Each exposure in our study is an indicator of LUTS, and the most common cause for LUTS in elderly men is prostatic enlargement due to BPH, but LUTS may also have other causes, so LUTS is not synonymous with BPH. However, a large proportion of men who undergo a TUR-P have obstructive symptoms due to prostatic enlargement, and in general these men have more severe symptoms and larger prostates than men who are pharmaceutically treated [3, 21]. Many guidelines recommend 5-ARI as treatment for LUTS in men with increased prostate volume, whereas α-blockers are recommended for LUTS in men with smaller prostates [22–24]. Based on these relationships between prostate volume, symptoms, and treatment modality, we assumed that TUR-P is the strongest indicator of BPH, followed by 5-ARI and α-blockers. TUR-P and α-blockers do not alter the endocrine milieu, whereas 5-ARI strongly suppress circulating levels of DHT by 70–90 % and increase testosterone levels by 25 % but do not alter estrogen levels, thus increasing the ratio of testosterone to estrogens [4]. Men with Klinefelter’s syndrome have a decreased ratio of testosterone to estrogens [25] and an increased risk of breast cancer [14, 26]. Obese men have lower levels of testosterone and lower ratio of testosterone to estrogens [27, 28]. Obesity is a risk factor for both male breast cancer [11, 14, 17] and BPH [29, 30].

We suggest that our observation of an increased risk of breast cancer in men with BPH who had undergone a TUR-P is due to a decreased ratio of testosterone to estrogen. In line with this speculation, the increased ratio caused by 5-ARI in men with BPH could be the mechanism behind the unaltered risk of male breast cancer in men with BPH on 5-ARI.

## Conclusion

We found no increase in the risk of breast cancer for men on 5-ARI, but there was an increased risk for men who had undergone a TUR-P, a strong indicator for BPH. Given the very low incidence of breast cancer and the null results from our cohort study and two large case–control studies, we suggest that the concern for breast cancer should not influence the selection of treatment for BPH.
